# National mortality trends in polyneuropathies and other disorders of the peripheral nervous system in the United States, 1999–2023: a CDC WONDER database analysis

**DOI:** 10.1186/s12883-026-04874-w

**Published:** 2026-04-07

**Authors:** Palwasha Asghar, Razeena Zahid, Safiullah Soomro, Arooj Ihsan Ullah, Sujata Lodh, Javeria Imran, Iftikhar Khan, Kamil Ahmad Kamil

**Affiliations:** 1 Khyber Medical College, Peshawar, Pakistan; 2 Services Institute of Medical Sciences, Lahore, Pakistan; 3 Dow Medical College, Karachi, Pakistan; 4 Fatima Jinnah Medical University, Lahore, Pakistan; 5 Dr. KNS Memorial Institute of Medical Sciences, Barabanki, India; 6 Shaheed Mohtarma Benazir Bhutto Medical College, Lyari, Karachi, Pakistan; 7 FMH College of Medicine and Dentistry, Lahore, Pakistan; 8 Internal Medicine Department, Mirwais Regional Hospital, Kandahar, Afghanistan

**Keywords:** Polyneuropathy, Peripheral nervous system disorders, Mortality trends, Age-adjusted mortality rate, CDC WONDER, United States

## Abstract

**Background:**

Peripheral neuropathies and other disorders of the peripheral nervous system carry a higher mortality risk due to respiratory failure, autonomic instability, and infections. The prevalence increases with age and due to other comorbid conditions. This study evaluates mortality trends in polyneuropathies and other disorders of the peripheral nervous system in the United States from 1999 to 2023.

**Methods:**

This study analyzed publicly available data from the CDC WONDER database (1999 to 2023) using ICD-10 codes for polyneuropathy and other disorders of the peripheral nervous system (G60-G64). Join point regression was used to compute annual percent changes and 95% CIs.

**Results:**

70,080 deaths were reported among individuals aged ≥ 55 years. Most deaths occurred at decedents’ homes (33.0%). AAMR increased from 3.18 in 1999 to 4.96 in 2023 (AAPC: 2.31%; 95% CI: 1.89–2.74%). Males maintained consistently higher AAMR than females. The greatest average CMR was demonstrated by those aged 76–85 + years (average CMR: 8.79 per 100,000 over the study period). Most deaths were observed in NH White individuals (88.5%), while Hispanic or Latino individuals had the lowest deaths (3.90%). The West exhibited the highest AAMR (4.20), and the lowest was observed in the Northeast (2.63). Substantial disparities were observed between metropolitan and non-metropolitan areas and across various states.

**Conclusion:**

Polyneuropathy related mortality has demonstrated an upward trend among adults aged ≥ 55 years from 1999 to 2023. significant inconsistencies persist with higher mortality among older adults, males, non-Hispanic white individuals, with regional disparities favoring west. This highlights the necessity of focused public health initiatives and enhanced care accessibility for all populations and areas that are at risk.

**Supplementary Information:**

The online version contains supplementary material available at 10.1186/s12883-026-04874-w.

## Introduction

The disorders of the peripheral nervous system, including polyneuropathies, are a diverse group of diseases characterized by diffuse or multifocal injury to peripheral nerves, leading to sensory, motor, and autonomic dysfunction [1]. These conditions have varying etiologies, such as autoimmune processes like Guillain-Barré syndrome (GBS), metabolic illnesses like diabetic neuropathy, toxic or drug-induced neuropathies, genetic neuropathies, and acute immune-mediated forms like chronic inflammatory demyelinating polyneuropathy (CIDP) [2, 3]. These conditions significantly increase the global healthcare burden, disability, and reduced quality of life [4].

Although many peripheral neuropathies are not directly fatal, higher risks of mortality persist due to consequences such as infections, respiratory failure, and autonomic instability, which can cause death, especially in patients with severe or systemic disease [5, 6]. The prevalence of peripheral neuropathies increases significantly with age, reaching about 8% in persons over 55. Together, they are thought to impact 2% to 3% of the general population [7]. The implication is worsened in the United States by the increasing prevalence of metabolic syndrome and diabetes, which together account for most cases of polyneuropathy [8]. Depending on the length of the disease and glycemic management, neuropathy can affect 28% to 50% of diabetics [9]. Recent population-based studies suggest that neuropathy is far from rare. In the United States, surveys of adults aged 40 and over have reported prevalence rates of 10.4% in middle-aged cohorts and up to 26.8% in seniors aged 70 and above [10]. The prevalence of chronic polyneuropathy is estimated to increase to approximately 7% in the older age group, and there are indications that prevalence may have been rising in recent decades [11]. Despite this growing burden, mortality data specific to polyneuropathies remain scarce, with limited population-level analyses describing temporal or demographic patterns across the United States.

Understanding these patterns is crucial for updating the healthcare policy and clinical practice. Identifying regions or populations with disproportionately high or increasing mortality rates could guide preventive intervention, resource allocation, and further investigation. Furthermore, long-term mortality monitoring may reveal the increasing impact of polyneuropathies in connection with comorbidities such as diabetes, autoimmune disorders, or toxic exposures. This study supports the need to quantify temporal trends, regional disparities, and percentile-based state-level distributions in AAMR, contributing to the broader understanding of public health priorities related to peripheral nerve disorders, and aims to assess the combined mortality trends of polyneuropathies and other disorders of the peripheral nervous system in the United States from 1999 to 2023. To assist its management and public health measures, we examine mortality patterns and geographic variations using the Wide-ranging Online Data for Epidemiologic Research (CDC WONDER) database maintained by the U.S. Centers for Disease Control and Prevention.

## Methods

### Study setting and population

The International Statistical Classification of Diseases and Related Health Problems-10th Revision (ICD-10) codes G60-G64 were used in this descriptive study to examine death certificate data from 1999 to 2023 for mortality associated with polyneuropathies and other disorders of the peripheral nervous system. Data were obtained from the CDC WONDER (Centers for Disease Control and Prevention Wide-ranging Online Data for Epidemiologic Research) database. We analyzed Multiple Cause of Death (MCOD) data, where any mention of G60-G64 on the death certificate was included, as peripheral neuropathies are often listed as contributing conditions rather than the underlying cause of death. The CDC WONDER system, a comprehensive and nationally representative data source, gathers death certificate records from all 51 states and the District of Columbia.

We restricted analysis to ages ≥ 55 years because deaths among younger individuals accounted for < 5% of total cases (*n* = 3,487) and were frequently suppressed by CDC WONDER due to confidentiality requirements (counts < 10 are suppressed). A sensitivity analysis including all ages is provided.

### Data extraction

The data from the Population size, place of death, year, demographics, area, state, and urban-rural classification were extracted. Sex, age, race/ethnicity, and medical facilities (inpatient, outpatient, emergency room, dead on arrival), the deceased’s residence, hospice facility, nursing home/long-term care, and other factors were among the demographics. Non-Hispanic (NH), NH Hispanic or Latino, NH Black or African American, NH American Indian or Alaskan Native, and NH Asian or Pacific Islander were the categories for the race/ethnicity. This data, which has been utilized in earlier CDC Wonder database analysis, is based on published data on death certificates. The CDC WONDER Database does not contain data for Asians and Non-Hispanic Americans. It made it impossible to perform an analysis on both. Based on the 2022 Census estimates and the 2023 Office of management and Budget Delineation of metropolitan and micropolitan statistical areas, countries were divided into six levels using the National Centre for Health Statistics ( NCHS) 2023 urban-rural classification scheme: four metropolitan(large central metro, large fringe metro, medium metro, and small metro), and non-two metropolitan(micropolitan and noncore). According to the U.S. Census Bureau, regions were classified as Northeast, Midwest, South, and West. Urban-rural data were available through 2020 due to a lag in the National Center for Health Statistics (NCHS) urban-rural classification scheme updates; the 2023 classification was not available in CDC WONDER at the time of analysis.

### Statistical analysis: age-specific

Age-adjusted mortality rates (AAMRs) per 100,000 population were computed annually from 1999 to 2023 by sex, race/ethnicity, state, and urban-rural status with 95% confidence intervals (CIs) to investigate national trends in mortality associated with polyneuropathy and other disorders of the peripheral nervous system. Age-adjusted mortality rates were calculated using age-specific rates standardized to the 2000 U.S. population with 10-year age strata (55–64, 65–74, 75–84, 85 + years). CDC WONDER suppresses counts < 10 for confidentiality; suppressed values were excluded from rate calculations and are noted as ‘Suppressed’ in tables. The Join Point Regression Program (Join Point V 5.4.0, National Cancer Institute) 12 was utilized to compute the annual percentage change with 95% confidence intervals (CIs) in age-adjusted mortality rates (AAMRs) to quantify national annual trends in mortality related to polyneuropathies and other disorders of the Peripheral Nervous System. Log-linear regression models are applied to the data; this technique finds times when statistically significant changes in AAMRs’ trends take place. APCs were deemed rising or decreasing using two-tailed t-testing, if the slope indicating the change in mortality was substantially different from zero; a p-value threshold of less than 0.05 indicated statistical significance.

## Results

**Fig. 1:**
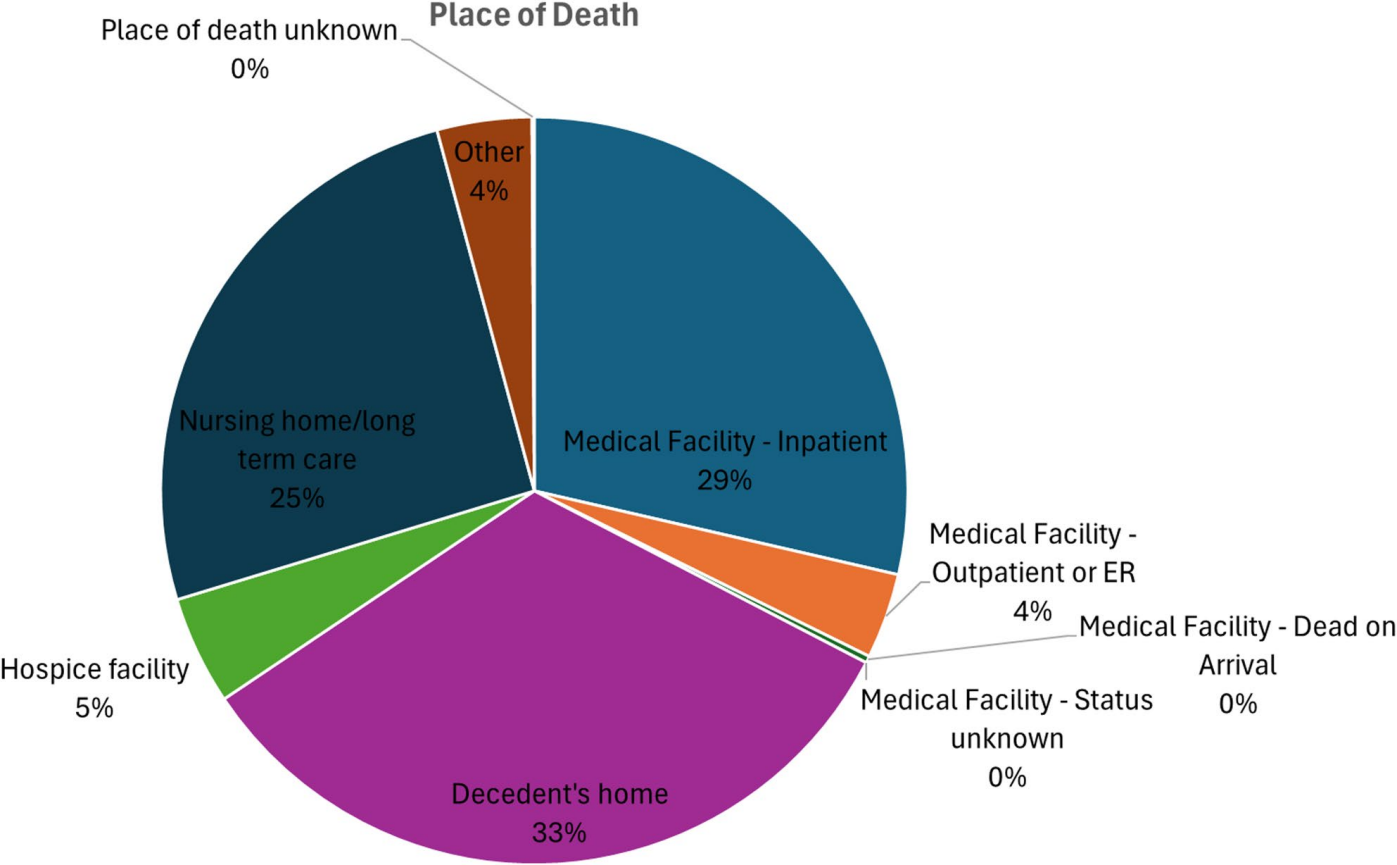
Distribution of polyneuropathy and other disorder-related deaths by place of occurrence in the United States, 1999–2023

A total of 70,080 deaths occurred in patients with polyneuropathies and other disorders of the peripheral nervous system aged ≥ 55 years from 1999 to 2023 in the United States, according to CDC WONDER data (Supplementary Table 1). Of the total 73,567 deaths across all ages, 70,080 (95.3%) occurred in individuals aged ≥ 55 years, while only 3,487 (4.7%) occurred in younger adults. Most of the deaths (33.0%) occurred at the decedent’s home, followed by medical facilities (32.6%), nursing homes (25.5%), hospice facilities (4.7%), and other places (4.1%) (Supplementary Table 2, Fig. 1). A higher proportion of deaths were recorded in males (53.4%) aged ≥ 76 years (62.9%) (Supplementary Table 1).

### Annual trends in patients with polyneuropathies and other disorders of the peripheral nervous system

**Fig. 2:**
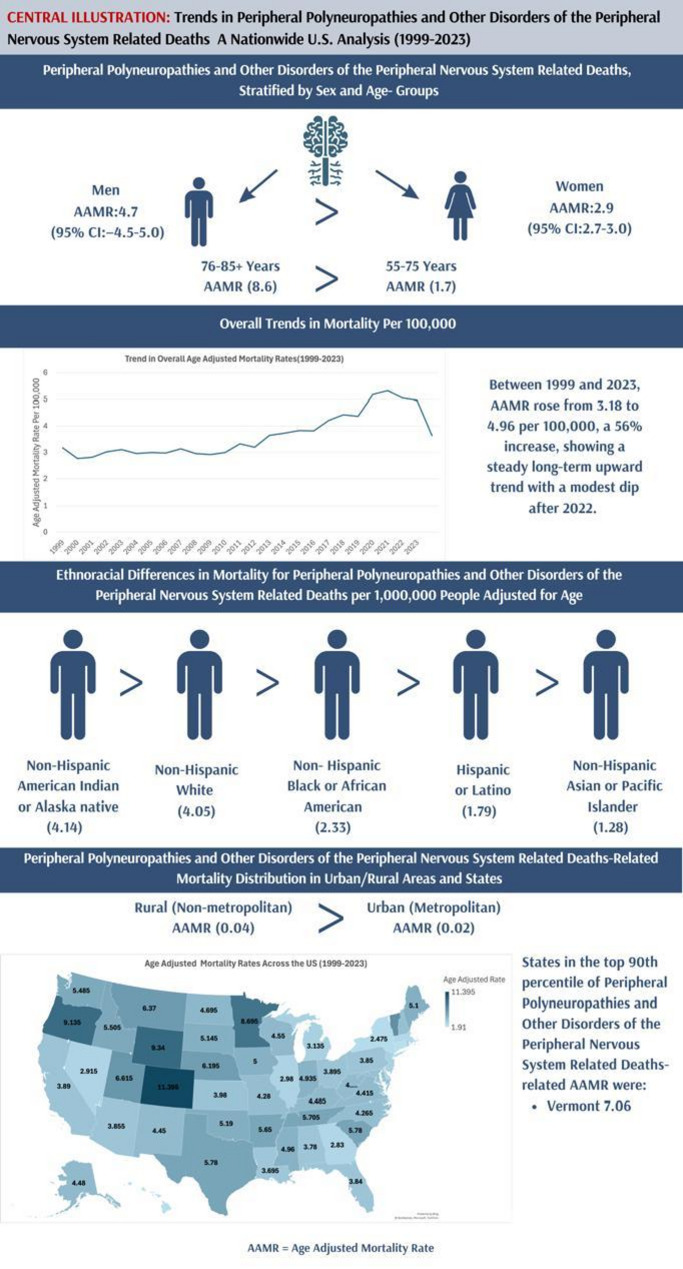
(Central Illustration): Trends and distribution of mortality from polyneuropathies and other disorders of the peripheral nervous system in the United States from 1999 to 2023, highlighting age-adjusted mortality patterns stratified by gender, race, urbanization, and location

**Fig. 3:**
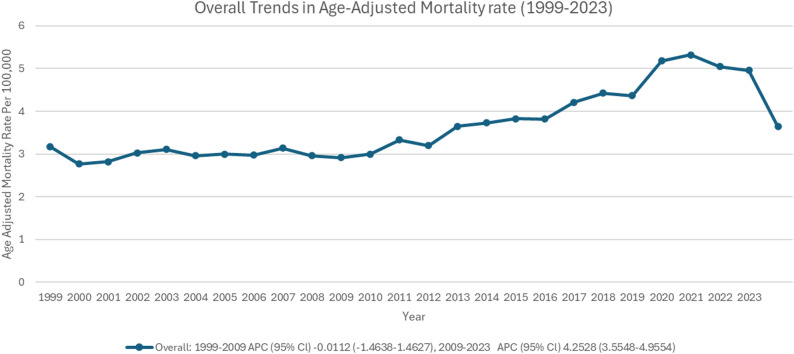
Overall trends of age-adjusted mortality rate in polyneuropathies and other disorders of the peripheral nervous system

The overall age-adjusted mortality rate (AAMR) in patients with polyneuropathies and other disorders of the peripheral nervous system increased from 3.18 (95% CI: 3.04 to 3.33) in 1999 to 4.96 (95% CI: 4.82 to 5.11) in 2023 (Supplementary Table 3, Figs. 2 and 3). The most significant rise was observed from 2009 to 2023 (APC: 4.25; 95% CI: 3.55 to 4.95; *p* < 0.001), following a nearly plateaued trend from 1999 to 2009 (APC: -0.01; 95% CI: -1.46 to 1.46). The average annual percent change (AAPC) over the entire study period was 2.31% (95% CI: 1.89–2.74%) (Supplementary Table 4, Fig. 3).

### Trends stratified by gender

**Fig. 4:**
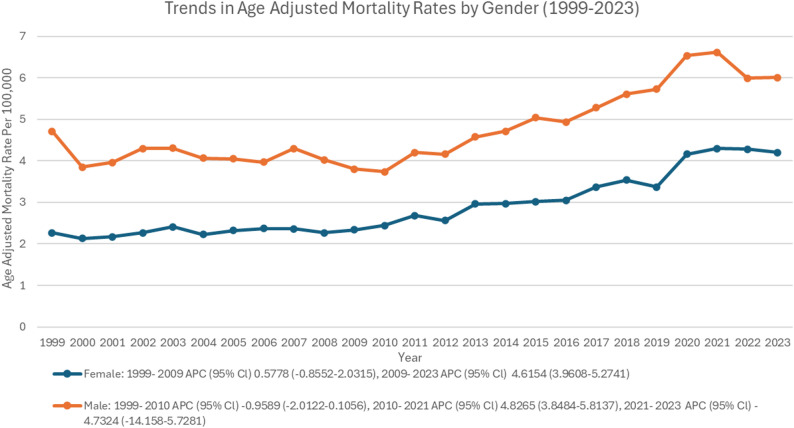
Trends of age-adjusted mortality rate in polyneuropathies and other disorders of the peripheral nervous system stratified by gender

In males, the AAMR slightly declined from 1999 to 2010 (APC: -0.95; 95% CI: -2.01 to 0.10), this decline was statistically not significant (*p* = 0.07) (Supplementary Table 3, Fig. 4). A significant rise was observed from 2010 to 2021 (APC: 4.82; 95% CI: 3.84 to 5.81; *p* < 0.001), followed by a non-significant decline from 2021 to 2023 (APC: -4.73; 95% CI: -14.15 to 5.72). In females, a significant rise was observed from 2009 to 2023 (APC: 4.62; 95% CI: 3.96 to 5.27; *p* < 0.001), preceded by a small non-significant rise from 1999 to 2009 (APC: 0.57; 95% CI: -0.85 to 2.03) (Supplementary Table 4, Fig. 4). The AAPC for males over the entire study period was 1.89% (95% CI: 1.21–2.58%), and for females was 2.67% (95% CI: 2.12–3.23%).

### Trends stratified by age

**Fig. 5:**
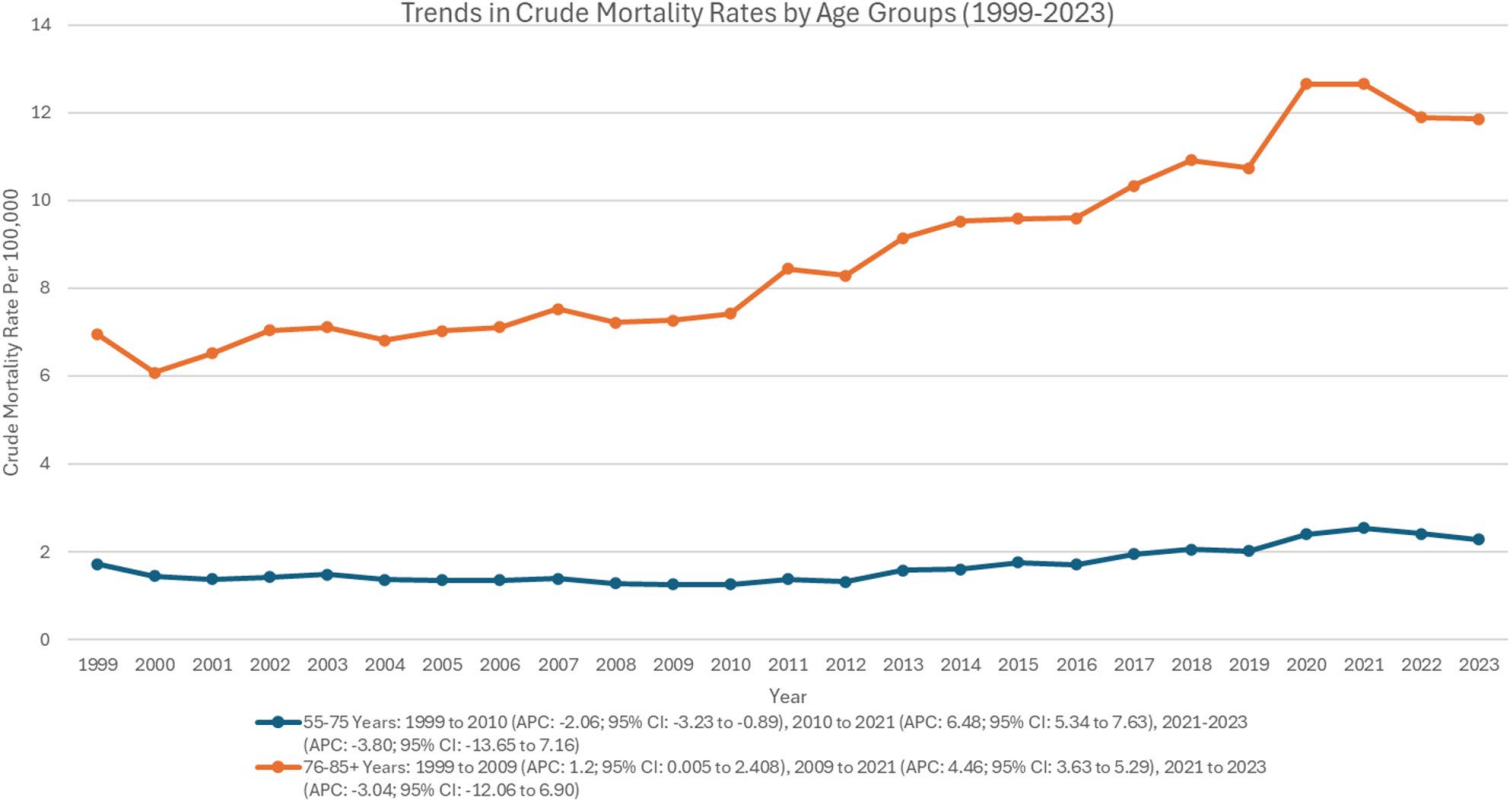
Trends of crude mortality rate in polyneuropathies and other disorders of the peripheral nervous system stratified by age

Mortality increased in most age categories, although consistency and steepness differed with age. The greatest crude mortality rate (CMR) was observed among those aged 76–85 + years, with an average CMR of 8.79 per 100,000 over the entire study period (1999–2023), followed by those aged 55–75 years (average CMR: 1.66) (Supplementary Table 5, Fig. 2). Among the age group of 55–75 years, a significant decline was noted from 1999 to 2010 (APC: -2.06; 95% CI: -3.23 to -0.89; *p* = 0.001), followed by a significant rise from 2010 to 2021 (APC: 6.48; 95% CI: 5.34 to 7.63; *p* < 0.001). From 2021 to 2023, a non-significant decline was observed (APC: -3.80; 95% CI: -13.65 to 7.16). Among the age group of 76–85 + years, a significant rise was observed from 1999 to 2009 (APC: 1.2; 95% CI: 0.005 to 2.408; *p* < 0.05), followed by another significant rise from 2009 to 2021 (APC: 4.46; 95% CI: 3.63 to 5.29; *p* < 0.001). A gradual but non-significant decline was observed from 2021 to 2023 (APC: -3.04; 95% CI: -12.06 to 6.90) (Fig. 5).

### Trends Stratified by Race/Ethnicity

**Fig. 6:**
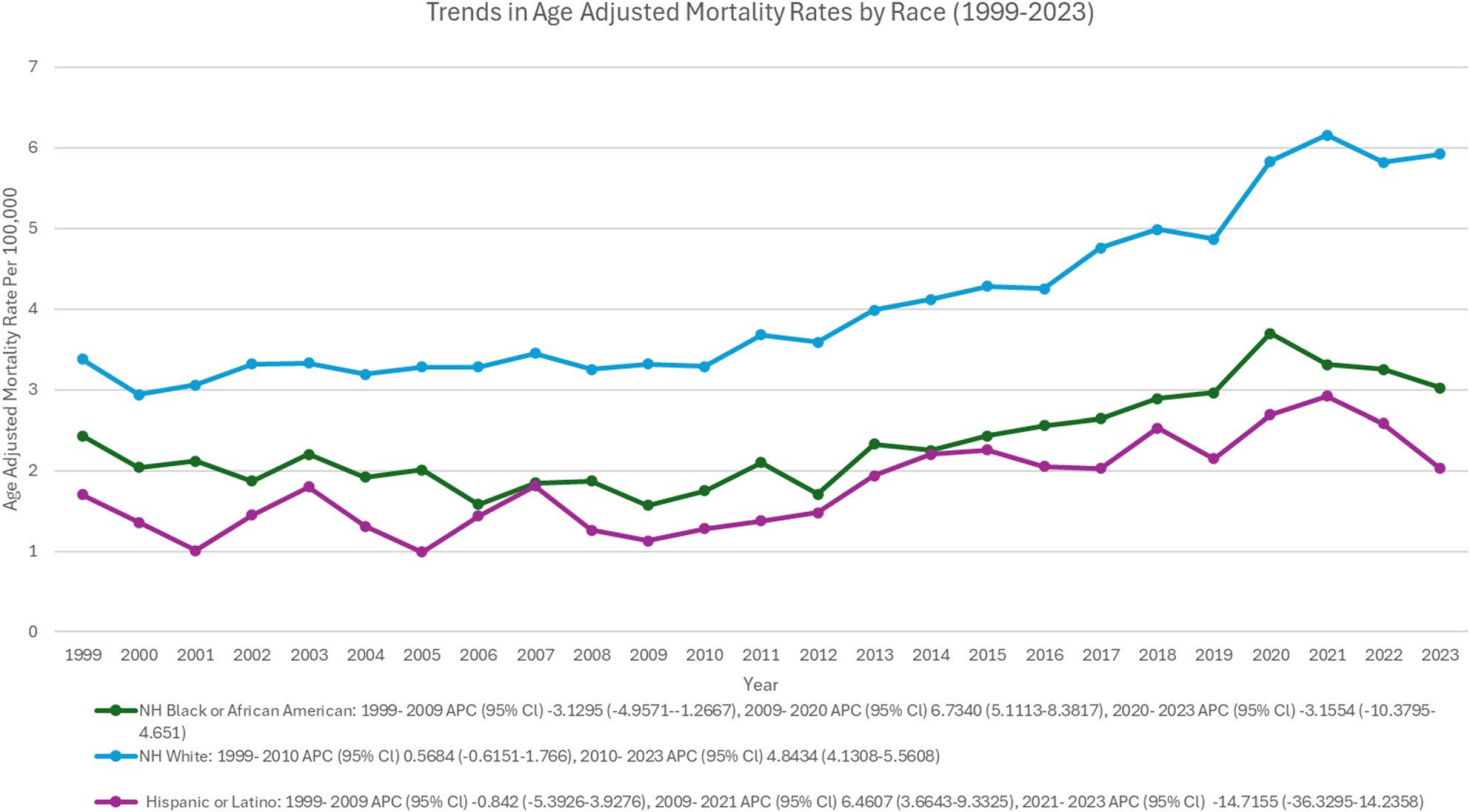
Trends of age-adjusted mortality rate in polyneuropathies and other disorders of the peripheral nervous system stratified by race/ethnicity

The non-Hispanic individuals accounted for the majority of deaths (96.0%), with white individuals comprising the largest proportion of deaths (88.5%). The Hispanics accounted for 3.90% of deaths (Supplementary Table 1). Black or African-American individuals observed a decline from 1999 to 2009 (APC: -3.12; 95% CI: -4.95 to -1.26; *p* < 0.05), a notable rise from 2009 to 2020 (APC: 6.73; 95% CI: 5.11 to 8.38; *p* < 0.001), and a slight non-significant decline from 2020 to 2023 (APC: -3.15; 95% CI: -10.3 to 4.65). Among White individuals, a slight non-significant rise from 1999 to 2010 (APC: 0.56; 95% CI: -0.61 to 1.76), followed by a steep upsurge from 2010 to 2023 (APC: 4.84; 95% CI: 4.13 to 5.56; *p* < 0.001) was observed. The Hispanic or Latino individuals exhibited a gradual decline from 1999 to 2009 (APC: -0.84; 95% CI: -5.39 to 3.92), a significant rise from 2009 to 2021 (APC: 6.46; 95% CI: 3.66 to 9.33; *p* < 0.001), an abrupt, non-significant decline from 2021 to 2023 (APC: -14.71; 95% CI: -36.32 to 14.23) (Supplementary Tables 4, 6, Fig. 6). The AAPC for NH White individuals was 2.43% (95% CI: 1.89–2.98%), for NH Black individuals was 2.12% (95% CI: 1.34–2.91%), and for Hispanic individuals was 1.87% (95% CI: 0.92–2.83%).

### Trends stratified by geographical region

**Fig. 7:**
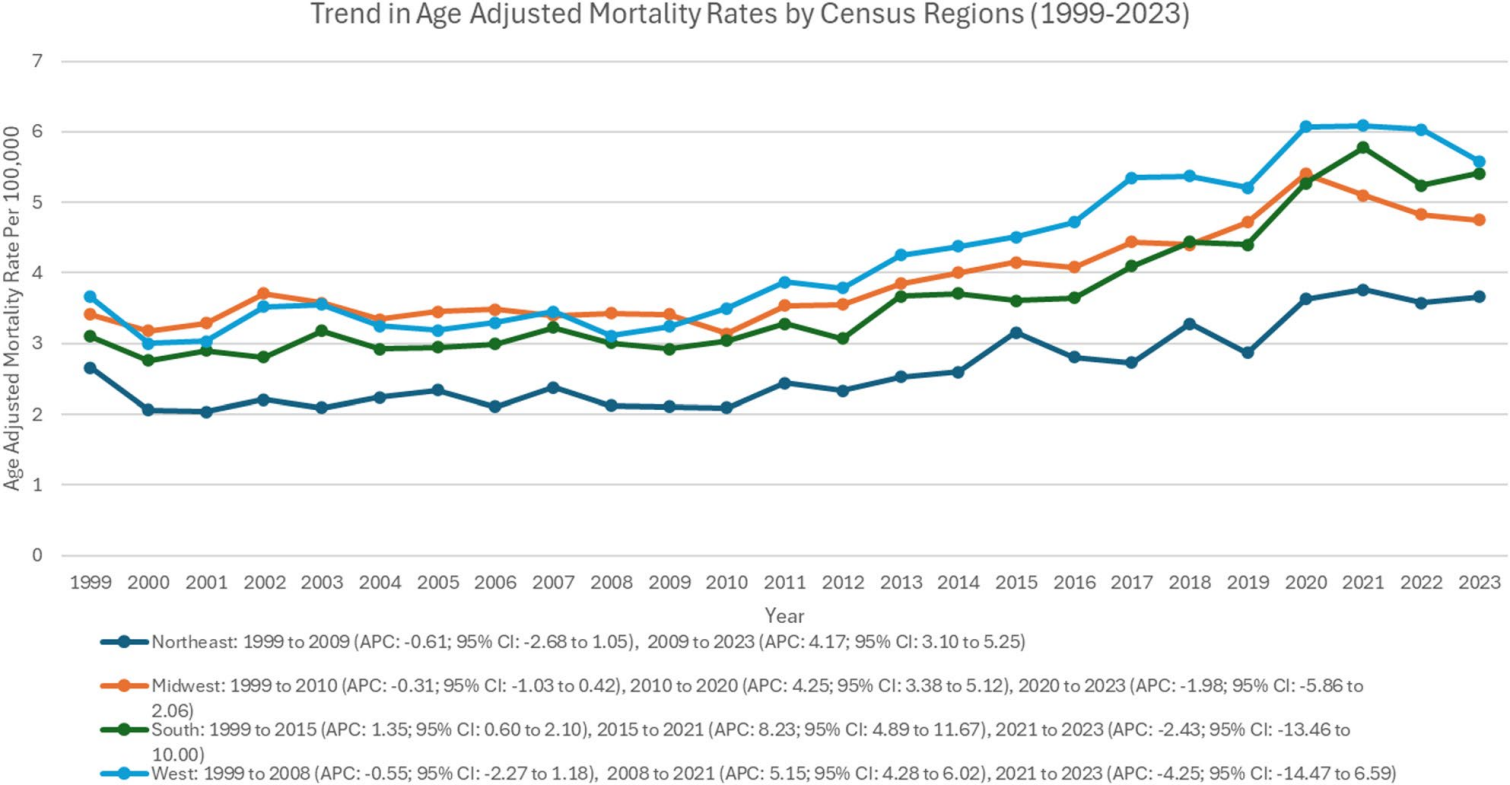
Trends of age-adjusted mortality rate in polyneuropathies and other disorders of the peripheral nervous system stratified by census region

The West exhibited the highest AAMR (4.20; 95% CI: 3.88 to 4.52), and the lowest was observed in the Northeast (2.63; 95% CI: 2.37 to 2.89) (Supplementary Table 7, Fig. 7). The Northeast exhibited a gradual, non-significant decline from 1999 to 2009 (APC: -0.61; 95% CI: -2.68 to 1.05), followed by a notable rise from 2009 to 2023 (APC: 4.17; 95% CI: 3.10 to 5.25; *p* < 0.001). The Midwest demonstrated a gradual decline from 1999 to 2010 (APC: -0.31; 95% CI: -1.03 to 0.42), a sharp rise from 2010 to 2020 (APC: 4.25; 95% CI: 3.38 to 5.12; *p* < 0.001), and a decline from 2020 to 2023 (APC: -1.98; 95% CI: -5.86 to 2.06). The South demonstrated a significant rise from 1999 to 2015 (APC: 1.35; 95% CI: 0.60 to 2.10; *p* < 0.05), followed by another significant rise from 2015 to 2021 (APC: 8.23; 95% CI: 4.89 to 11.67; *p* < 0.05), and a decline from 2021 to 2023 (APC: -2.43; 95% CI: -13.46 to 10.00), although the latter was not significant. The West exhibited a gradual, non-significant decline from 1999 to 2008 (APC: -0.55; 95% CI: -2.27 to 1.18), a significant rise from 2008 to 2021 (APC: 5.15; 95% CI: 4.28 to 6.02; *p* < 0.001), and another non-significant decline from 2021 to 2023 (APC: -4.25; 95% CI: -14.47 to 6.59) (Supplementary Table 7, supp Fig. 1).

Substantial disparities were observed across U.S. states, reflecting variations in demographics, healthcare access, and disease burden. The highest AAMR was observed in Colorado (11.4), and the lowest was observed in Hawaii (2.27). States falling within the top 90th percentile included Colorado (11.4), Wyoming (9.34), Oregon (9.14), Minnesota (8.70), and Vermont (7.06), which had overall higher AAMR than the states in the lower 10th percentile, which included Hawaii (2.27), Connecticut (2.28), New York (2.48), Delaware (2.64), and Massachusetts (2.68) (Supplementary Table 8, Fig. 2, supp Fig. 2).

**Fig. 8:**
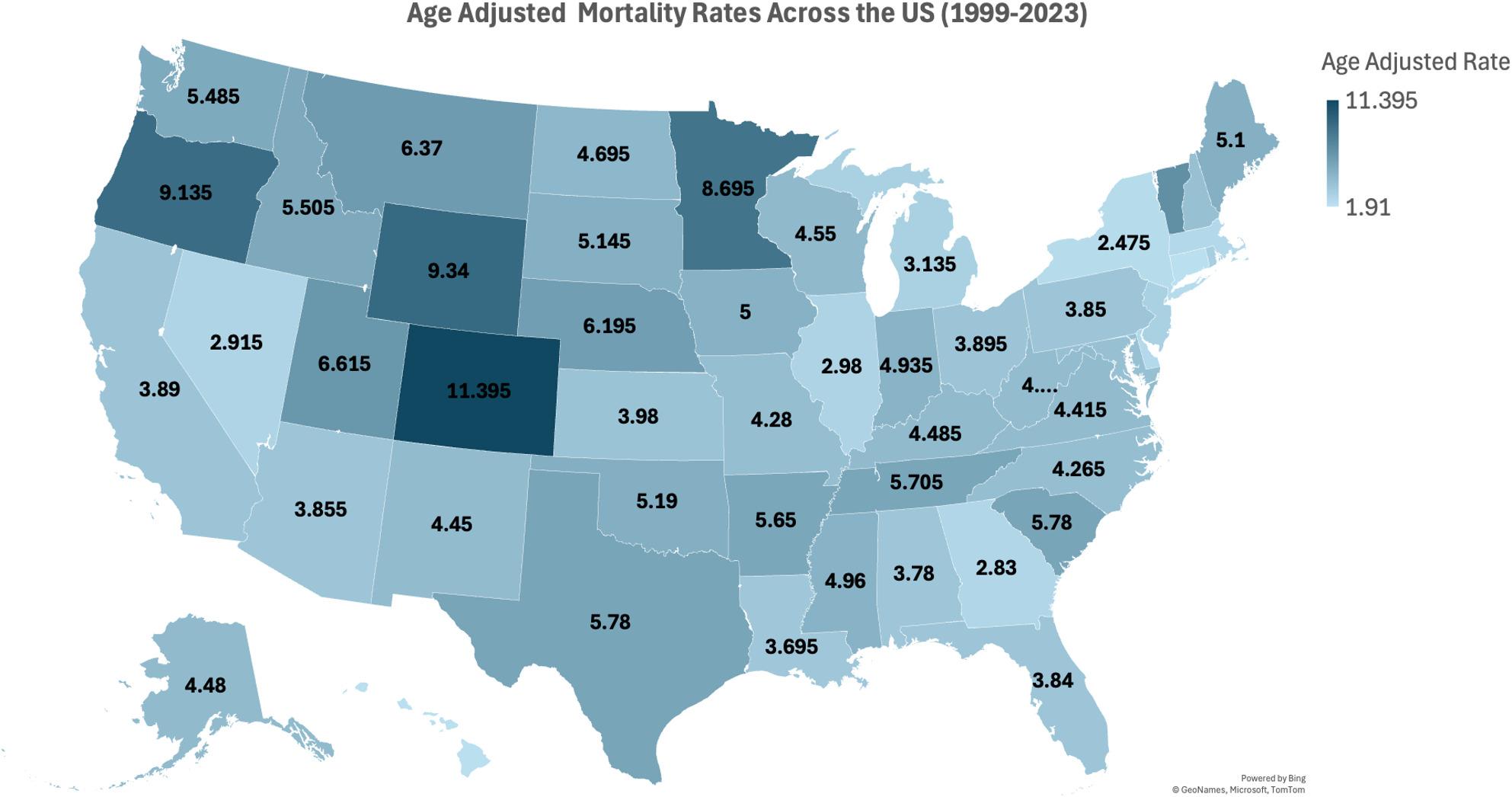
(Wonder map): Geographic distribution of age-adjusted mortality rates (AAMR) for polyneuropathies and other disorders of the peripheral nervous system across U.S. states, 1999–2023, based on CDC WONDER data. Darker shades indicate higher mortality rates

**Fig. 9:**
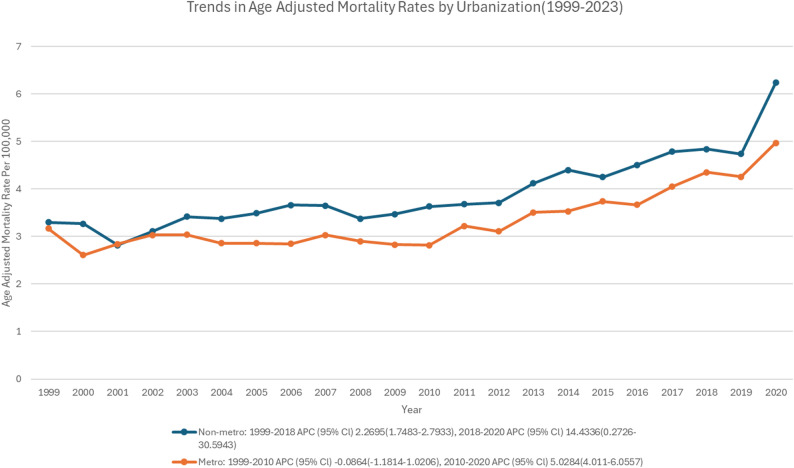
Trends of age-adjusted mortality rate in polyneuropathies and other disorders of the peripheral nervous system stratified by urbanization

Non-metropolitan areas had consistently higher AAMR than metropolitan areas across the study period (Fig. 2). Both areas experienced an overall increase in age-adjusted rates. Non-metropolitan areas exhibited a nearly plateaued trend from 1999 to 2010 (APC: -0.08; 95% CI: -1.18 to 1.02; *p* = 0.87), followed by a significant rise from 2010 to 2020 (APC: 5.02; 95% CI: 4.01 to 6.05; *p* < 0.001). Metropolitan areas demonstrated a gradual, significant rise from 1999 to 2018 (APC: 2.27; 95% CI: 1.74 to 2.79; *p* < 0.001), followed by a steep upsurge from 2018 to 2020 (APC: 14.43; 95% CI: 0.27 to 30.59; *p* < 0.05) (Supplementary Table 9, supp Fig. 3) ( Figs. 8 and 9).

## Discussion

Mortality rates for polyneuropathies and other peripheral nervous system (PNS) disorders have steadily increased over the past 2 decades in the United States with around 70,000 deaths from 1999 to 2023 according to our analysis. The overall increase in AAMR from 3.18 to 4.96 from 1999 to 2023 indicates the significant contribution of PNS disorders, which have received little attention historically, to neurological mortality. This trend is consistent with findings in recent epidemiological studies conducted in the United States, indicating the emergence of peripheral neuropathies as a public health concern, especially in older and multimorbid populations. It has been linked to diabetes, chronic kidney disease, alcohol use, chemotherapy, and idiopathic degeneration [11, 12, 13]. Improved diagnostic sensitivity and a greater recognition of neuropathy as a cause of death on certificates as a result of ICD-10 revisions and improved reporting frameworks coincide with the sharp increase after 2009 [14]. Additionally, the neurological burden of peripheral degeneration may have been revealed by population ageing, multimorbidity, and survival from other chronic diseases [15, 16].

Established sex-based differences in mortality may reflect higher prevalence of metabolic syndrome, alcohol-related neuropathies, and diabetes complications among males [17]. The steady increase in females after 2009 might be related to improved survival from chronic illnesses (such as diabetes and autoimmune disorders), allowing neuropathic complications to develop later in life [7], as well as the aging of the female population. Estrogenic neuroprotection and varying mitochondrial resiliency factors that diminished after menopause, are the mediators of sex differences in peripheral nerve degeneration [18, 19].

As anticipated, mortality was highest among those aged 76 years (83.9%), which is in line with the buildup of risk factors and the aging-related decrease in peripheral nerve regenerative capacity [20]. The sharp rise after 2010 is consistent with the ageing of the American population and the rise in multimorbidity. Older adults are especially vulnerable to drug-induced neuropathies (e.g., statins, chemotherapy, antibiotics), which are exacerbated by age-related oxidative stress, sarcopenia, and polypharmacy [21, 22].Competing mortality during the COVID-19 pandemic, which temporarily decreased reporting of non-COVID chronic neurological deaths, may have contributed to the brief decline after 2021 [23].

Hispanics and Blacks experienced the largest proportional increases after 2009, although non-Hispanic Whites accounted for 88.5% of deaths. These differences are a reflection of social determinants of health as well as disease biology. More advanced neuropathy presentations are a result of higher rates of diabetes, chronic infections, and delayed access to neurology services among minority populations [24, 25]. Additionally, research shows that racial minorities are underdiagnosed with peripheral neuropathies because of disparities in primary care access, unconscious bias, and uneven electrophysiologic testing utilization [26]. Although there has been a general improvement, mortality convergence after 2020 might also be a result of COVID-19’s disproportionate impact on these populations, which interferes with the management of chronic diseases [27].

Regional variations in environmental exposures, healthcare systems, and health-related behaviors are reflected in the West’s highest AAMR and the Northeast’s lowest. Despite their high rankings, states like Colorado and Oregon have ageing populations and longer survival rates from metabolic diseases, which leads to the accumulation of neuropathic sequelae [28]. On the other hand, the South’s high rates through 2021 are probably caused by socioeconomic hardship, a lack of access to healthcare, and a greater incidence of diabetes and obesity [29]. The West region’s highest AAMR (4.20) was disproportionately influenced by outlier states such as Colorado (11.4), Oregon (9.14), and Wyoming (9.34). This may reflect state-level differences in death certification practices, access to neurologic care, or true differences in disease burden requiring further investigation. For example, Colorado’s high rate might be related to its older population demographics and longer survival from chronic diseases, while state-specific registries or coding practices could also contribute to these variations.

The clear rural-urban divide, where non-metropolitan areas consistently have higher AAMRs, is consistent with a larger body of research showing that neurologists are harder to reach, rehabilitation services are underutilized, and diagnoses are delayed outside of metropolitan areas [28, 30]. These gaps were momentarily widened by the COVID-19 era because rural populations still had limited access to telehealth [31].

## Conclusion

The steady increase over several decades suggests extensive systemic deficiencies in long-term disease management and preventive neurology. Diabetes, renal failure, and nutritional deficiencies are common causes of peripheral neuropathies, which go undetected until they become severe. There is currently no national screening for subclinical neuropathy in high-risk adults, despite advancements in electrophysiology and neuroimaging [32].

Furthermore, despite medical advancements, the reduction of mortality for chronic neurological conditions has been limited due to underfunded rehabilitation and palliative frameworks [33]. Either better patient preference alignment or gaps in palliative support for neurodegenerative diseases are reflected in the finding that one-third of deaths happened at home [34].

### Limitations

This study has several limitations. First, because peripheral neuropathy is frequently coded as secondary to diabetes or renal failure, mortality data from death certificates may underestimate the burden of neuropathy due to misclassification, under-reporting, or attribution bias [35]. Second, the evaluation of comorbidities, etiology (such as diabetic versus idiopathic), or treatment status is restricted by the lack of clinical detail in CDC WONDER. Third, state-level estimates may be unstable due to small numbers in some strata and should be interpreted with caution.

Fourth, the ICD-10 codes G60-G64 encompass a heterogeneous group of disorders, including hereditary neuropathies (G60), inflammatory polyneuropathies (G61), toxic and diabetic neuropathies (G62), neuropathies in diseases classified elsewhere (G63), and unspecified disorders (G64). These entities differ fundamentally in etiology, prognosis, and affected populations. Combining them may mask distinct trends within subcategories; however, the small number of deaths in individual subcodes precluded separate trend analysis.

Fifth, urban-rural data were only available through 2020 due to lags in NCHS classification updates, limiting assessment of recent trends in these areas. Finally, while we identified temporal trends and demographic disparities, causal inferences cannot be drawn from death certificate data alone.

Despite these limitations, large-scale surveillance offers an essential perspective on long-term population trends.

### Public health and clinical implications

This persistent increase highlights the necessity of:

Prevention at the population level, focusing on risk factors that can be changed, like obesity, diabetes, and alcohol consumption.

Enhanced availability of neurology services for diagnosis and rehabilitation in areas with limited resources and outside of major cities.

Research on regenerative and neuroprotective treatments, especially for age-related neuropathies.

Fair laws that address racial and socioeconomic inequalities in the treatment of chronic neurological conditions.

Peripheral neuropathy should be given priority in ageing and public health frameworks as neurological mortality continues to shift from acute diseases (like stroke) to chronic neurodegeneration [6, 35].

## Supplementary Information

Supplementary Material 1.

Supplementary Material 2.

## Data Availability

The dataset analyzed in this study is publicly available on the CDC WONDER online database ( https://wonder.cdc.gov/ ). No special access permissions were required.

## Abbreviations

AAMR: Age-Adjusted Mortality Rate
APC: Annual Percent Change
AAPC: Average Annual Percent Change
CDC: Centers for Disease Control and Prevention
ICD-10: International Classification of Diseases, 10th Revision
NH: Non-Hispanic
