# Supplementary material for: National mortality trends in polyneuropathies and other disorders of the peripheral nervous system in the United States, 1999–2023: a CDC WONDER database analysis
Source: BMC Neurol. 2026 Apr 7;26:325. doi: 10.1186/s12883-026-04874-w (PMC13182005; doi:10.1186/s12883-026-04874-w)
Supplement: Supplementary file 1 — Supplementary Material 1. [file 12883_2026_4874_MOESM1_ESM.docx]

**Supplemental Table 1:** Polyneuropathies and other Disorders of the Peripheral Nervous System related Deaths, Stratified by Sex and Race, in Adults in the United States, 1999 to 2023.

| Year | Overall | Women | Men | NH American Indian or Alaska Native | NH Asian or Pacific Islander | NH Black or African American | NH White | Hispanic or Latino | Population |
| --- | --- | --- | --- | --- | --- | --- | --- | --- | --- |
| 1999 | 1867 | 798 | 1069 | 12 | 10 | 116 | 1680 | 46 | 58575867 |
| 2000 | 1627 | 747 | 880 | Suppressed | 14 | 101 | 1466 | 40 | 59266437 |
| 2001 | 1698 | 777 | 921 | Suppressed | 11 | 102 | 1547 | 29 | 60395586 |
| 2002 | 1846 | 830 | 1016 | Suppressed | Suppressed | 94 | 1691 | 46 | 62225539 |
| 2003 | 1921 | 891 | 1030 | Suppressed | 16 | 114 | 1725 | 57 | 63872474 |
| 2004 | 1848 | 842 | 1006 | Suppressed | 17 | 102 | 1673 | 49 | 65508623 |
| 2005 | 1918 | 889 | 1029 | Suppressed | 17 | 112 | 1746 | 38 | 67291295 |
| 2006 | 1967 | 927 | 1040 | Suppressed | 15 | 88 | 1793 | 57 | 69094220 |
| 2007 | 2098 | 945 | 1153 | Suppressed | 14 | 105 | 1889 | 78 | 70954145 |
| 2008 | 2017 | 919 | 1098 | 10 | 15 | 110 | 1819 | 56 | 72934684 |
| 2009 | 2044 | 980 | 1064 | Suppressed | 16 | 98 | 1870 | 51 | 75028775 |
| 2010 | 2109 | 1040 | 1069 | 14 | 18 | 109 | 1905 | 61 | 76750713 |
| 2011 | 2427 | 1173 | 1254 | Suppressed | 28 | 138 | 2177 | 73 | 79456281 |
| 2012 | 2410 | 1134 | 1276 | 10 | 31 | 118 | 2166 | 80 | 81731558 |
| 2013 | 2803 | 1354 | 1449 | 18 | 32 | 164 | 2471 | 115 | 84020505 |
| 2014 | 2954 | 1395 | 1559 | 21 | 39 | 171 | 2588 | 131 | 86320792 |
| 2015 | 3137 | 1430 | 1707 | 16 | 35 | 195 | 2730 | 154 | 88638671 |
| 2016 | 3180 | 1460 | 1720 | 26 | 56 | 205 | 2748 | 140 | 90707339 |
| 2017 | 3589 | 1674 | 1915 | 19 | 52 | 216 | 3147 | 150 | 92854337 |
| 2018 | 3888 | 1794 | 2094 | 13 | 44 | 251 | 3383 | 194 | 94703829 |
| 2019 | 3914 | 1741 | 2173 | 19 | 82 | 261 | 3370 | 176 | 96506800 |
| 2020 | 4728 | 2188 | 2540 | 25 | 86 | 337 | 4052 | 224 | 98063042 |
| 2021 | 4747 | 2181 | 2566 | 21 | 57 | 299 | 4090 | 255 | 98651017 |
| 2022 | 4681 | 2291 | 2390 | 15 | 63 | 295 | 4049 | 232 | 99880289 |
| 2023 | 4662 | 2232 | 2430 | 16 | 46 | 281 | 4096 | 193 | 101102772 |
| Total | **70080** | **32632** | **37448** | **255** | **814** | **4182** | **61871** | **2725** | **1994535590** |

***NH = Non hispanic**

**Supplemental Table 2:** Polyneuropathies and other Disorders of the Peripheral Nervous System related Deaths, Stratified by Place of Death, in Adults in the United States, 1999 to 2023.

| Year | Medical Facility | Nursing Home/Long-term care Facility | Hospices | Home |
| --- | --- | --- | --- | --- |
| 1999 | 952 | 496 | Missing | 382 |
| 2000 | 832 | 454 | Missing | 294 |
| 2001 | 812 | 472 | Missing | 364 |
| 2002 | 885 | 491 | Missing | 418 |
| 2003 | 889 | 498 | Suppressed | 462 |
| 2004 | 836 | 485 | Suppressed | 446 |
| 2005 | 828 | 524 | 11 | 473 |
| 2006 | 801 | 553 | 22 | 520 |
| 2007 | 868 | 534 | 42 | 569 |
| 2008 | 797 | 539 | 44 | 533 |
| 2009 | 769 | 534 | 68 | 574 |
| 2010 | 731 | 560 | 80 | 637 |
| 2011 | 797 | 702 | 95 | 726 |
| 2012 | 775 | 668 | 114 | 753 |
| 2013 | 865 | 724 | 151 | 953 |
| 2014 | 868 | 845 | 134 | 985 |
| 2015 | 930 | 862 | 192 | 1034 |
| 2016 | 910 | 837 | 185 | 1112 |
| 2017 | 984 | 920 | 250 | 1276 |
| 2018 | 1015 | 994 | 290 | 1434 |
| 2019 | 1012 | 1004 | 310 | 1414 |
| 2020 | 1147 | 1146 | 286 | 1922 |
| 2021 | 1249 | 951 | 333 | 2003 |
| 2022 | 1192 | 959 | 353 | 1937 |
| 2023 | 1112 | 1100 | 363 | 1882 |
| Total | 22856 | 17852 | 3323 | 23103 |

**Supplemental Table 3: Overall and Sex Stratified Polyneuropathies**

**and other Disorders of the Peripheral Nervous System Related**

**Age-Adjusted Mortality Rates per 100,000 in Adults in the United States,**

**1999 to 2023**

| Year | Overall | Men | Women |
| --- | --- | --- | --- |
| 1999 | **3.18 (3.04-3.33)** | **2.27 (2.11-2.43)** | **4.71 (4.42-5.00)** |
| 2000 | **2.77 (2.63-2.90)** | **2.13 (1.97-2.28)** | **3.85 (3.59-4.10)** |
| 2001 | **2.82 (2.69-2.95)** | **2.17 (2.02-2.33)** | **3.96 (3.70-4.22)** |
| 2002 | **3.03 (2.89-3.16)** | **2.27 (2.12-2.43)** | **4.30 (4.03-4.57)** |
| 2003 | **3.11 (2.97-3.25)** | **2.41 (2.25-2.57)** | **4.31 (4.04-4.57)** |
| 2004 | **2.96 (2.82-3.09)** | **2.23 (2.08-2.38)** | **4.07 (3.81-4.33)** |
| 2005 | **3.00 (2.86-3.13)** | **2.32 (2.17-2.47)** | **4.05 (3.80-4.30)** |
| 2006 | **2.98 (2.85-3.12)** | **2.37 (2.22-2.52)** | **3.97 (3.72-4.21)** |
| 2007 | **3.14 (3.00-3.27)** | **2.36 (2.21-2.52)** | **4.30 (4.04-4.55)** |
| 2008 | **2.96 (2.83-3.09)** | **2.27 (2.12-2.42)** | **4.03 (3.78-4.27)** |
| 2009 | **2.92 (2.79-3.05)** | **2.34 (2.19-2.49)** | **3.80 (3.56-4.03)** |
| 2010 | **3.00 (2.87-3.12)** | **2.44 (2.29-2.59)** | **3.74 (3.51-3.97)** |
| 2011 | **3.33 (3.20-3.47)** | **2.68 (2.52-2.84)** | **4.20 (3.96-4.43)** |
| 2012 | **3.20 (3.07-3.33)** | **2.56 (2.41-2.71)** | **4.16 (3.93-4.39)** |
| 2013 | **3.65 (3.51-3.78)** | **2.96 (2.80-3.12)** | **4.58 (4.34-4.82)** |
| 2014 | **3.73 (3.59-3.86)** | **2.97 (2.81-3.13)** | **4.71 (4.47-4.95)** |
| 2015 | **3.83 (3.70-3.97)** | **3.02 (2.86-3.18)** | **5.04 (4.80-5.28)** |
| 2016 | **3.82 (3.69-3.96)** | **3.05 (2.89-3.21)** | **4.94 (4.70-5.17)** |
| 2017 | **4.21 (4.07-4.35)** | **3.37 (3.20-3.53)** | **5.28 (5.04-5.53)** |
| 2018 | **4.43 (4.29-4.57)** | **3.54 (3.37-3.70)** | **5.61 (5.36-5.85)** |
| 2019 | **4.37 (4.23-4.50)** | **3.37 (3.21-3.53)** | **5.73 (5.49-5.98)** |
| 2020 | **5.18 (5.03-5.33)** | **4.16 (3.98-4.33)** | **6.54 (6.28-6.79)** |
| 2021 | **5.32 (5.17-5.47)** | **4.30 (4.12-4.48)** | **6.62 (6.36-6.89)** |
| 2022 | **5.05 (4.90-5.19)** | **4.28 (4.10-4.46)** | **5.99 (5.75-6.24)** |
| 2023 | **4.96 (4.82-5.11)** | **4.20 (4.03-4.37)** | **6.01 (5.76-6.25)** |

------------------------------------------------------------------------

**Supplemental Table 4:** Annual percent change (APC) of Polyneuropathies and other Disorders of the Peripheral Nervous System Age-Adjusted Mortality Rates per 100,000 in Adults in the United States, 1999 to 2023.

| Category | Year Interval | APC (95% CI) | AAPC (95% CI) |
| --- | --- | --- | --- |
| Overall | 1999-2009 | -0.0112 (-1.4638-1.4627) | 2.45 (1.76-3.15) |
|  | 2009-2023 | 4.2528 (3.5548-4.9554) |  |
| Men | 1999-2010 | -0.9589 (-2.0122-0.1056) | 1.32 (0.31-2.35) |
|  | 2010-2021 | 4.8265 (3.8484-5.8137) |  |
|  | 2021-2023 | -4.7324 (-14.158-5.7281) |  |
| Women | 1999-2009 | 0.5778 (-0.8552-2.0315) | 2.91 (2.24-3.59) |
|  | 2009-2023 | 4.6154 (3.9608-5.2741) |  |
| NH Black or African American | 1999-2009 | -3.1295 (-4.9571--1.2667) | 1.27 (-0.07-2.63) |
|  | 2009-2020 | 6.7340 (5.1113-8.3817) |  |
|  | 2020-2023 | -3.1554 (-10.3795-4.651) |  |
| NH White | 1999-2010 | 0.5684 (-0.6151-1.766) | 2.86 (2.23-3.50) |
|  | 2010-2023 | 4.8434 (4.1308-5.5608) |  |
| Hispanic or Latino | 1999-2009 | -0.842 (-5.3926-3.9276) | 1.46 (-1.69-4.71) |
|  | 2009-2021 | 6.4607 (3.6643-9.3325) |  |
|  | 2021-2023 | -14.7155 (-36.3295-14.2358) |  |
| Metropolitan areas | 1999-2010 | -0.0864 (-1.1814-1.0206) | 2.32 (1.62-3.02) |
|  | 2010-2020 | 5.0284 (4.011-6.0557) |  |
| Non-metropolitan areas | 1999-2018 | 2.2695 (1.7483-2.7933) | 3.37 (2.09-4.67) |
|  | 2018-2020 | 14.4336 (0.2726-30.5943) |  |
| Northeast | 1999-2009 | -0.6123 (-2.683-1.5025) | 2.15 (1.14-3.18) |
|  | 2009-2023 | 4.1765 (3.1078-5.2563) |  |
| Midwest | 1999-2010 | -0.3063 (-1.0359-0.4286) | 1.35 (0.70-2.01) |
|  | 2010-2020 | 4.2502 (3.3843-5.1233) |  |
|  | 2020-2023 | -1.9815 (-5.8672-2.0647) |  |
| South | 1999-2015 | 1.3512 (0.6042-2.1037) | 2.70 (1.41-4.01) |
|  | 2015-2021 | 8.2321 (4.8992-11.6709) |  |
|  | 2021-2023 | -2.4334 (-13.4689-10.0096) |  |
| West | 1999-2008 | -0.5596 (-2.2721-1.1828) | 2.15 (1.01-3.30) |
|  | 2008-2021 | 5.1542 (4.2882-6.0275) |  |
|  | 2021-2023 | -4.5218 (-14.4765-6.5916) |  |

| **Year Interval** | **APC (95% CI)** |
| --- | --- |

**Supplemental Table 5:** Polyneuropathies and other Disorders of the Peripheral Nervous System related Age-Adjusted Mortality Rates per 100,000 and Crude rate, Stratified by Age-Groups in Adults in the United States, 1999 to 2023.

|  | Age 55-75 Years |  | Age 76-85+ years |  |
| --- | --- | --- | --- | --- |
| Year | **Age Adjusted Rate** | **Crude Rate** | **Age Adjusted Rate** | **Crude Rate** |
| 1999 | 1.68 (1.55-1.8) | 1.72(1.6-1.85) | 7.01 (6.6-7.41) | 6.96(6.56-7.36) |
| 2000 | 1.45 (1.33-1.56) | 1.45(1.33-1.56) | 6.13 (5.75-6.5) | 6.08(5.7-6.45) |
| 2001 | 1.36 (1.25-1.47) | 1.37(1.26-1.48) | 6.53 (6.15-6.92) | 6.52(6.13-6.9) |
| 2002 | 1.45 (1.33-1.56) | 1.42(1.31-1.53) | 7.04 (6.64-7.43) | 7.04(6.64-7.44) |
| 2003 | 1.55 (1.43-1.66) | 1.48(1.37-1.59) | 7.09 (6.69-7.49) | 7.11(6.71-7.5) |
| 2004 | 1.45 (1.33-1.56) | 1.36(1.26-1.47) | 6.79 (6.4-7.17) | 6.81(6.43-7.2) |
| 2005 | 1.42 (1.31-1.52) | 1.35(1.25-1.45) | 7.01 (6.62-7.4) | 7.03(6.64-7.42) |
| 2006 | 1.4 (1.3-1.51) | 1.35(1.25-1.35) | 7 (6.61-7.38) | 7.11(6.72-7.5) |
| 2007 | 1.46 (1.35-1.57) | 1.39(1.29-1.49) | 7.39 (7-7.79) | 7.53(7.13-7.93) |
| 2008 | 1.36 (1.26-1.46) | 1.28(1.18-1.37) | 7.04 (6.66-7.42) | 7.22(6.83-7.61) |
| 2009 | 1.32 (1.22-1.41) | 1.25(1.16-1.34) | 7 (6.62-7.38) | 7.26(6.88-7.65) |
| 2010 | 1.36 (1.26-1.46) | 1.26(1.17-1.35) | 7.15 (6.77-7.53) | 7.42(7.02-7.81) |
| 2011 | 1.46 (1.36-1.56) | 1.37(1.28-1.47) | 8.08 (7.68-8.48) | 8.44(8.02-8.85) |
| 2012 | 1.37 (1.28-1.47) | 1.31(1.22-1.4) | 7.84 (7.45-8.22) | 8.29(7.88-8.7) |
| 2013 | 1.66 (1.56-1.76) | 1.58(1.49-1.68) | 8.69 (8.28-9.1) | 9.14(8.72-9.57) |
| 2014 | 1.66 (1.56-1.76) | 1.6(1.5-1.7) | 8.97 (8.56-9.38) | 9.53(9.1-9.96) |
| 2015 | 1.79 (1.69-1.89) | 1.75(1.65-1.85) | 9.02 (8.61-9.42) | 9.58(9.16-10.01) |
| 2016 | 1.75 (1.65-1.85) | 1.71(1.62-1.81) | 9.09 (8.69-9.5) | 9.6(9.17-10.02) |
| 2017 | 1.99 (1.89-2.09) | 1.95(1.85-2.06) | 9.85 (9.43-10.27) | 10.34(9.9-10.77) |
| 2018 | 2.09 (1.98-2.2) | 2.05(1.95-2.16) | 10.39 (9.97-10.81) | 10.92(10.48-11.35) |
| 2019 | 2.03 (1.93-2.14) | 2.02(1.91-2.12) | 10.29 (9.88-10.7) | 10.74(10.31-11.17) |
| 2020 | 2.42 (2.31-2.53) | 2.4(2.29-2.52) | 12.21 (11.76-12.65) | 12.66(12.2-13.12) |
| 2021 | 2.52 (2.41-2.63) | 2.54(2.42-2.65) | 12.43 (11.97-12.89) | 12.65(12.19-13.12) |
| 2022 | 2.42 (2.31-2.53) | 2.41(2.3-2.52) | 11.73 (11.3-12.16) | 11.89(11.45-12.32) |
| 2023 | 2.22 (2.11-2.32) | 2.28(2.18-2.39) | 11.94 (11.51-12.37) | 11.86(11.43-12.29) |

**Supplemental Table 6**: Polyneuropathies and other Disorders of the Peripheral Nervous System related Deaths, Stratified by Race, in Adults in the United States, 1999 to 2023.

| Year | NH White | NH Black or African American | NH American Indian or Alaska Native | Hispanic or Latino | NH Asian or Pacific Islander |
| --- | --- | --- | --- | --- | --- |
| 1999 | 3.38(3.22-3.55) | 2.43(1.99-2.88) | Unreliable (2.79-9.99) | 1.7(1.24-2.28) | Unreliable (0.37-1.42) |
| 2000 | 2.94(2.79-3.09) | 2.04(1.64-2.44) | Suppressed | 1.36(096-1.87) | Unreliable (0.62-1.99) |
| 2001 | 3.06(2.91-3.21) | 2.12(1.71-2.54) | Suppressed | 1.01(0.67-1.47) | Unreliable (0.37-1.42) |
| 2002 | 3.32(3.16-3.47) | 1.87(1.5-2.29) | Suppressed | 1.45(1.05-1.95) | Suppressed |
| 2003 | 3.33(3.17-3.49) | 2.2(1.79-2.61) | Suppressed | 1.8(1.335-2.35) | Unreliable (0.52-1.54) |
| 2004 | 3.19(3.03-3.34) | 1.92(1.54-2.3) | Suppressed | 1.31(0.96-1.75) | Unreliable (0.53-1.5) |
| 2005 | 3.28(3.13-3.44) | 2.01(1.63-2.39) | Suppressed | 0.99(0.69-1.38) | Unreliable (0.52-1.47) |
| 2006 | 3.28(3.13-3.44) | 1.58(1.26-1.96) | Suppressed | 1.44(1.08-1.88) | Unreliable (0.41-1.25) |
| 2007 | 3.45(3.29-3.61) | 1.85(1.48-2.21) | Suppressed | 1.81(1.41-2.28) | Unreliable (0.37-1.18) |
| 2008 | 3.25(3.1-3.4) | 1.87(1.51-2.23) | Unreliable (1.51-6.27) | 1.26(0.94-1.65) | Unreliable (0.41-1.2) |
| 2009 | 3.32(3.17-3.47) | 1.57(1.27-1.92) | Suppressed | 1.13(0.83-1.5) | Unreliable (0.4-1.18) |
| 2010 | 3.29(3.14-3.44) | 1.75(1.41-2.09) | Unreliable (2.34-7.52) | 1.28(0.97-1.65) | 1.09(0.45-1.19) |
| 2011 | 3.68(3.53-3.84) | 2.1(1.74-2.46) | Suppressed | 1.38(1.07-1.75) | 1.02(0.67-1.49) |
| 2012 | 3.59(3.43-3.74) | 1.71(1.39-2.03) | Unreliable (0.85-3.54) | 1.48(1.16-1.85) | 1.16(0.78-1.65) |
| 2013 | 3.99(3.83-4.14) | 2.33(1.96-2.7) | Unreliable (2.26-6.42) | 1.94(1.58-2.31) | 1.04(0.71-1.47) |
| 2014 | 4.12(3.96-4.28) | 2.25(1.9-2.6) | 4.72(2.84-7.38) | 2.2(1.81-2.58) | 1.19(0.84-1.63) |
| 2015 | 4.28(4.12-4.44) | 2.43(2.08-2.78) | Unreliable (2.17-6.41) | 2.26(1.89-2.63) | 1.02(0.7-1.41) |
| 2016 | 4.25(4.09-4.41) | 2.56(2.2-2.92) | 4.19(2.71-6.18) | 2.05(1.7-2.39) | 1.46(1.1-1.91) |
| 2017 | 4.76(4.59-4.93) | 2.64(2.28-3) | Unreliable (2.12-5.83) | 2.03(1.69-2.36) | 1.35(1.01-1.78) |
| 2018 | 4.99(4.82-5.16) | 2.89(2.52-3.26) | Unreliable (1.11-3.74) | 2.53(2.16-2.89) | 1.11(0.8-1.49) |
| 2019 | 4.87(4.71-5.04) | 2.96(2.59-3.34) | Unreliable (2.04-5.45) | 2.15(1.82-2.47) | 1.93(1.53-2.4) |
| 2020 | 5.83(5.65-6.01) | 3.7(3.29-4.11) | 3.93(2.49-5.89) | 2.69(2.33-3.05) | 1.8(1.44-2.23) |
| 2021 | 6.16(5.97-6.35) | 3.31(2.92-3.7) | 3.72(2.27-5.74) | 2.92(2.55-3.29) | 1.29(0.97-1.67) |
| 2022 | 5.82(5.64-6) | 3.25(2.86-3.63) | Unreliable (1.41-4.34) | 2.58(2.24-2.92) | 1.34(1.03-1.72) |
| 2023 | 5.92(5.74-6.1) | 3.02(2.65-3.38) | Unreliable (1.43-4.2) | 2.03(1.74-2.33) | 0.91(0.66-1.22) |
|  |  |  |  |  |  |

*NH = non-Hispanic.

**Supplemental Table 7**: Polyneuropathies and other Disorders of the Peripheral Nervous System related Deaths, Stratified by Census Region, in Adults in the United States, 1999 to 2023.

| Census Region | Year | Age-Adjusted Rate (95% CI) |
| --- | --- | --- |
| Northeast | 1999 | 2.66(2.37-2.94) |
| Northeast | 2000 | 2.06(1.81-2.31) |
| Northeast | 2001 | 2.04(1.79-2.29) |
| Northeast | 2002 | 2.21(1.95-2.46) |
| Northeast | 2003 | 2.09(1.84-2.34) |
| Northeast | 2004 | 2.24(1.99-2.5) |
| Northeast | 2005 | 2.34(2.08-2.6) |
| Northeast | 2006 | 2.11(1.86-2.35) |
| Northeast | 2007 | 2.38(2.12-2.64) |
| Northeast | 2008 | 2.12(1.88-2.37) |
| Northeast | 2009 | 2.11(1.87-2.36) |
| Northeast | 2010 | 2.09(1.85-2.33) |
| Northeast | 2011 | 2.44(2.19-2.7) |
| Northeast | 2012 | 2.33(2.08-2.58) |
| Northeast | 2013 | 2.53(2.27-2.79) |
| Northeast | 2014 | 2.6(2.34-2.85) |
| Northeast | 2015 | 3.16(2.88-3.44) |
| Northeast | 2016 | 2.81(2.54-3.07) |
| Northeast | 2017 | 2.73(2.48-2.99) |
| Northeast | 2018 | 3.28(3-3.57) |
| Northeast | 2019 | 2.87(2.61-3.13) |
| Northeast | 2020 | 3.63(3.34-3.92) |
| Northeast | 2021 | 3.76(3.46-4.05) |
| Northeast | 2022 | 3.58(3.3-3.87) |
| Northeast | 2023 | 3.66(3.37-3.94) |
| Midwest | 1999 | 3.41(3.11-3.72) |
| Midwest | 2000 | 3.18(2.88-3.48) |
| Midwest | 2001 | 3.29(2.99-3.59) |
| Midwest | 2002 | 3.71(3.4-4.03) |
| Midwest | 2003 | 3.58(3.27-3.88) |
| Midwest | 2004 | 3.34(3.04-3.63) |
| Midwest | 2005 | 3.45(3.16-3.75) |
| Midwest | 2006 | 3.48(3.18-3.78) |
| Midwest | 2007 | 3.4(3.1-3.69) |
| Midwest | 2008 | 3.43(3.14-3.73) |
| Midwest | 2009 | 3.41(3.13-3.7) |
| Midwest | 2010 | 3.14(2.86-3.41) |
| Midwest | 2011 | 3.54(3.25-3.83) |
| Midwest | 2012 | 3.55(3.26-3.84) |
| Midwest | 2013 | 3.85(3.56-4.15) |
| Midwest | 2014 | 4(3.7-4.3) |
| Midwest | 2015 | 4.15(3.85-4.45) |
| Midwest | 2016 | 4.08(3.78-4.38) |
| Midwest | 2017 | 4.44(4.13-4.75) |
| Midwest | 2018 | 4.4(4.1-4.7) |
| Midwest | 2019 | 4.72(4.41-5.03) |
| Midwest | 2020 | 5.4(5.07-5.72) |
| Midwest | 2021 | 5.1(4.78-5.43) |
| Midwest | 2022 | 4.83(4.53-5.14) |
| Midwest | 2023 | 4.75(4.44-5.05) |
| South | 1999 | 3.1(2.86-3.34) |
| South | 2000 | 2.76(2.54-2.99) |
| South | 2001 | 2.9(2.67-3.13) |
| South | 2002 | 2.81(2.58-3.03) |
| South | 2003 | 3.18(2.94-3.41) |
| South | 2004 | 2.92(2.69-3.14) |
| South | 2005 | 2.95(2.73-3.17) |
| South | 2006 | 2.99(2.77-3.21) |
| South | 2007 | 3.23(3-3.46) |
| South | 2008 | 3.01(2.79-3.23) |
| South | 2009 | 2.92(2.71-3.13) |
| South | 2010 | 3.04(2.83-3.25) |
| South | 2011 | 3.28(3.06-3.49) |
| South | 2012 | 3.07(2.86-3.28) |
| South | 2013 | 3.67(3.44-3.9) |
| South | 2014 | 3.71(3.48-3.93) |
| South | 2015 | 3.61(3.39-3.83) |
| South | 2016 | 3.65(3.44-3.87) |
| South | 2017 | 4.1(3.88-4.32) |
| South | 2018 | 4.44(4.21-4.67) |
| South | 2019 | 4.4(4.17-4.62) |
| South | 2020 | 5.27(5.02-5.51) |
| South | 2021 | 5.78(5.52-6.04) |
| South | 2022 | 5.24(5-5.48) |
| South | 2023 | 5.41(5.16-5.65) |
| West | 1999 | 3.66(3.31-4.01) |
| West | 2000 | 3(2.69-3.31) |
| West | 2001 | 3.03(2.72-3.35) |
| West | 2002 | 3.52(3.19-3.86) |
| West | 2003 | 3.55(3.22-3.88) |
| West | 2004 | 3.25(2.94-3.56) |
| West | 2005 | 3.19(2.88-3.49) |
| West | 2006 | 3.3(3-3.61) |
| West | 2007 | 3.45(3.14-3.76) |
| West | 2008 | 3.11(2.82-3.4) |
| West | 2009 | 3.24(2.95-3.53) |
| West | 2010 | 3.5(3.19-3.8) |
| West | 2011 | 3.87(3.56-4.18) |
| West | 2012 | 3.79(3.48-4.09) |
| West | 2013 | 4.25(3.94-4.57) |
| West | 2014 | 4.38(4.06-4.69) |
| West | 2015 | 4.51(4.19-4.82) |
| West | 2016 | 4.72(4.41-5.04) |
| West | 2017 | 5.35(5.01-5.68) |
| West | 2018 | 5.37(5.04-5.7) |
| West | 2019 | 5.21(4.89-5.53) |
| West | 2020 | 6.07(5.73-6.41) |
| West | 2021 | 6.09(5.74-6.44) |
| West | 2022 | 6.03(5.69-6.36) |
| West | 2023 | 5.58(5.26-5.9) |

**Supplemental Table 8**: Polyneuropathies and other Disorders of the Peripheral Nervous System related Deaths, Stratified by States, in Adults in the United States, 1999 to 2023.

| Age-Adjusted Rate (95% CI) | | |
| --- | --- | --- |
| State | **1999-2020 Age- Adjusted Rate (95% CI)** | **2021-2023 Age-Adjusted Rate (95% CI)** |
| Alabama | 3.76(3.52-4) | 3.8(3.22-4.38) |
| Alaska | 4.48(3.58-5.54) | 4.26(2.12-6.49) |
| Arizona | 2.99(2.81-3.18) | 4.72(4.19-5.25) |
| Arkansas | 3.91(3.6-4.21) | 7.39(6.34-8.44) |
| California | 3.84(3.74-3.93) | 3.94(3.71-4.16) |
| Colorado | 6.52(6.17-6.86) | 16.27(15.01-17.52) |
| Connecticut | 2.32(2.11-2.53) | 2.24(1.76-2.82) |
| Delaware | 2.59(2.14-3.04) | 2.68(1.75-3.93) |
| District of Columbia | 1.91(1.43-2.49) | Suppressed |
| Florida | 2.61(2.52-2.69) | 5.07(4.79-5.36) |
| Georgia | 2.55(2.39-2.71) | 3.11(2.71-3.52) |
| Hawaii | 2.23(1.89-2.56) | 2.31(1.59-3.25) |
| Idaho | 4.37(3.89-4.84) | 6.64(5.36-8.13) |
| Illinois | 3.15(3.01-3.29) | 2.81(2.49-3.13) |
| Indiana | 4.29(4.07-4.51) | 5.58(4.96-6.21) |
| Iowa | 4.09(3.79-4.38) | 5.91(5.01-6.81) |
| Kansas | 3.64(3.33-3.94) | 4.32(3.49-5.15) |
| Kentucky | 3.33(3.09-3.57) | 5.64(4.88-6.41) |
| Louisiana | 2.48(2.27-2.69) | 4.91(4.18-5.63) |
| Maine | 3.83(3.41-4.25) | 6.37(5.12-7.82) |
| Maryland | 3.3(3.08-3.51) | 4.95(4.34-5.57) |
| Massachusetts | 2.49(2.33-2.65) | 2.87(2.44-3.29) |
| Michigan | 2.7(2.56-2.84) | 3.57(3.17-3.96) |
| Minnesota | 5.87(5.59-6.16) | 11.52(10.55-12.49) |
| Mississippi | 3.35(3.05-3.65) | 6.57(5.54-7.6) |
| Missouri | 3.95(3.73-4.16) | 4.61(4.03-5.19) |
| Montana | 5.07(4.49-5.66) | 7.67(6.04-9.6) |
| Nebraska | 5.26(4.81-5.71) | 7.13(5.81-8.45) |
| Nevada | 1.8(1.56-2.04) | 4.03(3.26-4.92) |
| New Hampshire | 3.47(3.03-3.9) | 4.8(3.66-6.17) |
| New Jersey | 2.77(2.62-2.91) | 3.48(3.06-3.89) |
| New Mexico | 3.41(3.05-3.77) | 5.49(4.42-6.56) |
| New York | 1.59(1.51-1.67) | 3.36(3.09-3.63) |
| North Carolina | 3.77(3.59-3.94) | 4.76(4.3-5.23) |
| North Dakota | 4.41(3.76-5.07) | 4.98(3.36-7.12) |
| Ohio | 3.96(3.81-4.12) | 3.83(3.45-4.22) |
| Oklahoma | 4.45(4.15-4.75) | 5.93(5.07-6.79) |
| Oregon | 6.38(6.04-6.72) | 11.89(10.76-13.02) |
| Pennsylvania | 3.3(3.18-3.43) | 4.4(4.03-4.78) |
| Rhode Island | 3.4(2.95-3.85) | 3.14(2.13-4.45) |
| South Carolina | 4.55(4.27-4.82) | 7.01(6.22-7.8) |
| South Dakota | 4.34(3.75-4.92) | 5.95(4.34-7.96) |
| Tennessee | 4.42(4.19-4.66) | 6.99(6.28-7.69) |
| Texas | 4.61(4.47-4.74) | 6.95(6.57-7.34) |
| Utah | 5.12(4.67-5.58) | 8.11(6.77-9.46) |
| Vermont | 5.5(4.73-6.27) | 8.62(6.44-11.31) |
| Virginia | 3.32(3.13-3.5) | 5.51(4.95-6.07) |
| Washington | 4.55(4.32-4.78) | 6.42(5.77-7.06) |
| West Virginia | 3.71(3.36-4.07) | 5.06(4.05-6.24) |
| Wisconsin | 3.65(3.44-3.86) | 5.45(4.81-6.1) |
| Wyoming | 5.79(4.89-6.69) | 12.89(9.86-16.55) |

**Supplemental Table 9**: Polyneuropathies and other Disorders of the Peripheral Nervous System related Deaths, Stratified by Urban-Rural Classification in the United States, 1999 to 2020.

| Age-Adjusted Rate (95% CI) | | |
| --- | --- | --- |
| Year | **Metropolitan** | **Nonmetropolitan** |
| 1999 | 3.17(3.01-3.33) | 3.3(2.97-3.64) |
| 2000 | 2.61(2.47-2.76) | 3.27(2.94-3.6) |
| 2001 | 2.84(2.69-2.99) | 2.82(2.51-3.13) |
| 2002 | 3.03(2.87-3.18) | 3.11(2.79-3.43) |
| 2003 | 3.04(2.88-3.19) | 3.42(3.09-3.76) |
| 2004 | 2.86(2.71-3.01) | 3.38(3.05-3.72) |
| 2005 | 2.86(2.72-3.01) | 3.49(3.16-3.83) |
| 2006 | 2.85(2.71-3) | 3.66(3.31-4) |
| 2007 | 3.03(2.89-3.18) | 3.65(3.31-3.99) |
| 2008 | 2.9(2.76-3.04) | 3.38(3.05-3.7) |
| 2009 | 2.83(2.69-2.97) | 3.47(3.14-3.8) |
| 2010 | 2.82(2.68-2.95) | 3.63(3.3-3.97) |
| 2011 | 3.22(3.08-3.37) | 3.68(3.35-4.01) |
| 2012 | 3.11(2.97-3.25) | 3.71(3.38-4.04) |
| 2013 | 3.51(3.36-3.66) | 4.12(3.77-4.46) |
| 2014 | 3.53(3.39-3.68) | 4.4(4.04-4.75) |
| 2015 | 3.74(3.59-3.89) | 4.25(3.91-4.6) |
| 2016 | 3.67(3.52-3.81) | 4.51(4.16-4.86) |
| 2017 | 4.05(3.9-4.2) | 4.79(4.43-5.15) |
| 2018 | 4.35(4.2-4.51) | 4.84(4.49-5.2) |
| 2019 | 4.26(4.12-4.41) | 4.74(4.39-5.09) |
| 2020 | 4.97(4.81-5.13) | 6.24(5.84-6.64) |
