# Supplementary material for: National mortality trends in polyneuropathies and other disorders of the peripheral nervous system in the United States, 1999–2023: a CDC WONDER database analysis
Source: BMC Neurol. 2026 Apr 7;26:325. doi: 10.1186/s12883-026-04874-w (PMC13182005; doi:10.1186/s12883-026-04874-w)
Supplement: Supplementary file 2 — Supplementary Material 2. [file 12883_2026_4874_MOESM2_ESM.docx]

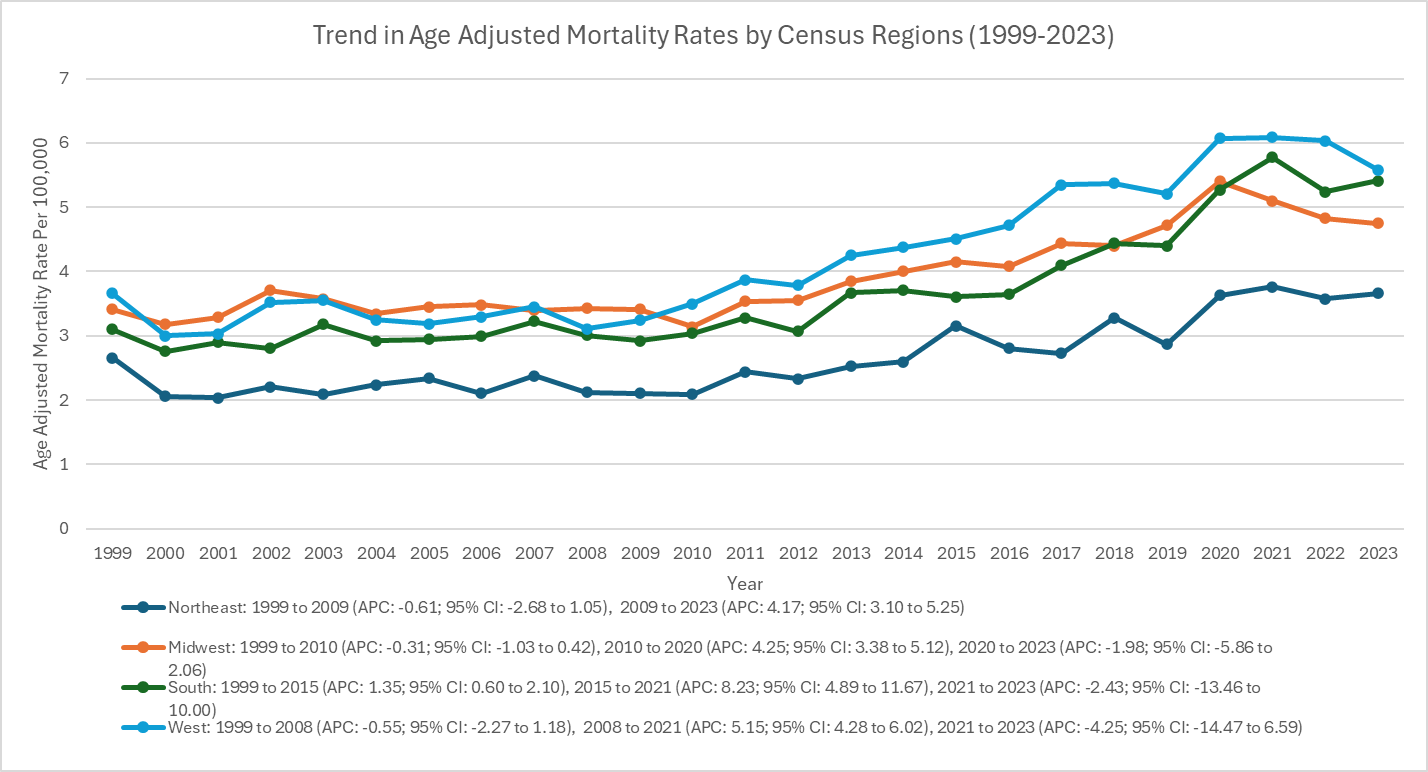


Supp Fig. 1. Trends of Age-Adjusted Mortality Rate in Polyneuropathies and Other Disorders of the Peripheral Nervous System Stratified by Census Region.


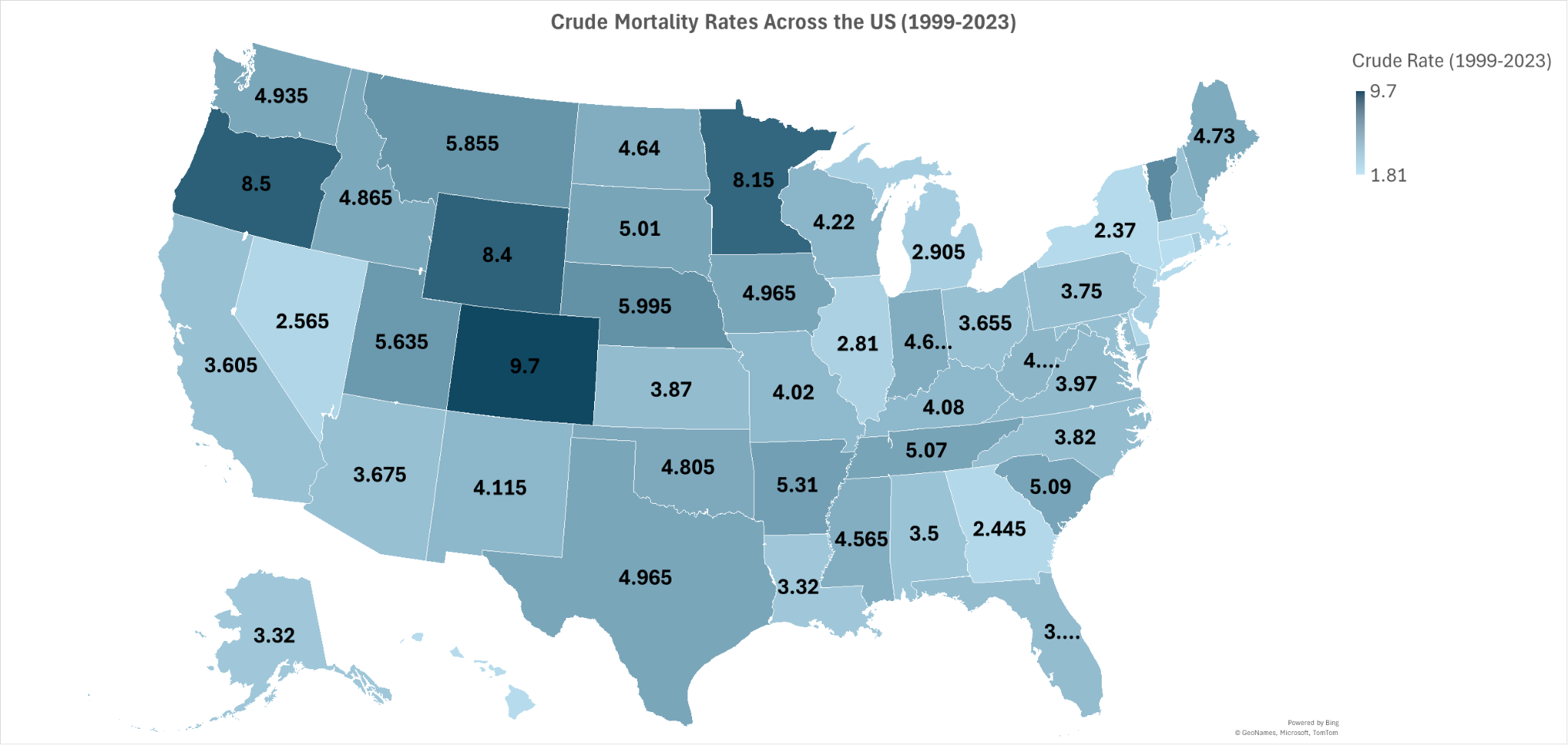


Supp Fig. 2. (Wonder map): Geographic distribution of age-adjusted mortality rates (AAMR) for polyneuropathies and other disorders of the peripheral nervous system across U.S. states, 1999–2023, based on CDC WONDER data. Darker shades indicate higher mortality rates.


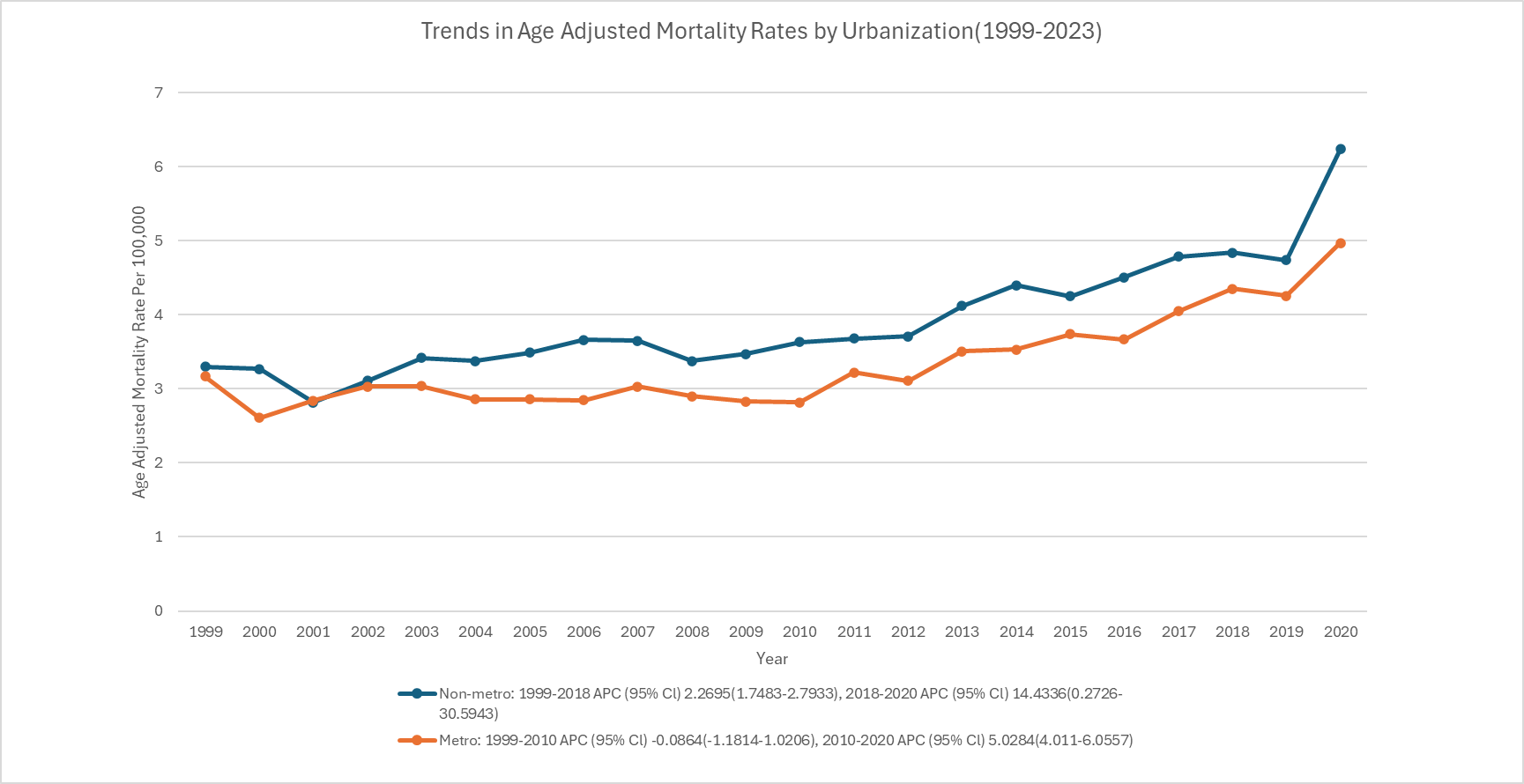


Supp Fig. 3. Trends of Age-Adjusted Mortality Rate in Polyneuropathies and Other Disorders of the Peripheral Nervous System Stratified by Urbanization.
